# Comparative transcriptome and metabolome analyses of cherry leaves spot disease caused by *Alternaria alternata*

**DOI:** 10.3389/fpls.2023.1129515

**Published:** 2023-02-09

**Authors:** Liu-Yi Pan, Jing Zhou, Yan Sun, Bai-Xue Qiao, Tian Wan, Rui-Quan Guo, Juan Zhang, Dong-Qian Shan, Yu-Liang Cai

**Affiliations:** ^1^College of Horticulture, Northwest A&F University, Yangling, Shaanxi, China; ^2^College of Horticulture and Forestry, Tarim University, Alar, Xinjiang, China

**Keywords:** transcriptome, metabolome, cherry leaves, *Alternaria alternata*, defensive response

## Abstract

*Alternaria alternata* is a necrotrophic fungal pathogen with a broad host range that causes widespread and devastating disease in sweet cherry (*Prunus avium*). We selected a resistant cultivar (RC) and a susceptible cultivar (SC) of cherry and used a combined physiological, transcriptomic, and metabolomic approach to investigate the molecular mechanisms underlying the plant’s resistance to *A. alternata*, of which little is known. We found that *A. alternata* infection stimulated the outbreak of reactive oxygen species (ROS) in cherry. The responses of the antioxidant enzymes and chitinase to disease were observed earlier in the RC than in the SC. Moreover, cell wall defense ability was stronger in the RC. Differential genes and metabolites involved in defense responses and secondary metabolism were primarily enriched in the biosynthesis of phenylpropanoids, tropane, piperidine and pyridine alkaloids, flavonoids, amino acids, and α-linolenic acid. Reprogramming the phenylpropanoid pathway and the α-linolenic acid metabolic pathway led to lignin accumulation and early induction of jasmonic acid signaling, respectively, in the RC, which consequently enhanced antifungal and ROS scavenging activity. The RC contained a high level of coumarin, and *in vitro* tests showed that coumarin significantly inhibited *A. alternata* growth and development and had antifungal effect on cherry leaves. In addition, differentially expressed genes encoding transcription factors from the MYB, NAC, WRKY, ERF, and bHLH families were highly expressed, they could be the key responsive factor in the response of cherry to infection by *A. alternata*. Overall, this study provides molecular clues and a multifaceted understanding of the specific response of cherry to *A. alternata*.

## 1 Introduction

The sweet cherry (*Prunus avium* L.) industry has expanded rapidly in recent years with the development of numerous cultivars and is of high economic importance in temperate regions worldwide (Wünsch and Hormaza, 2002). Despite the expansion of cherry cultivation areas, diseases caused by pests have become one of the main factors restricting advances in the cherry industry (Pasquariello et al., 2015). Cherry black spot is a common fungal disease caused by the pathogen *Alternaria alternata*, which mainly damages leaves and fruits (Yang et al., 2020; Pan et al., 2022). The disease occurs during the hot and rainy conditions in summer and autumn and can reach a rate of 60–100%. The severely diseased leaves all fall off their trees from August to September, directly affecting the tree’s vegetative growth post-harvest for that year, as well as flower bud differentiation and yield for the following year. The disease lesions are small black circular spots that gradually expand to 3–5 mm in diameter. These spots may coalesce or continue to expand, becoming irregular and darker in color (Thomidis and Tsipouridis, 2006). Black spot disease has been studied in several plants, including pear (Yang et al., 2015), citrus (Gai et al., 2019), and marigold (Cheng et al., 2019). However, research on cherry black spot disease is limited, with investigations focused on pathogen isolation, identification, and chemical control. Currently, the measures for preventing and controlling cherry diseases mainly include chemical pesticide application. Although these pesticides can reduce the probability of disease occurrence, they are environmental pollutants. Moreover, the various types and dosages of pesticides can result in drug-resistant pathogens, thus increasing the difficulty of control (Li et al., 2022). Therefore, a better understanding of the defense mechanism of cherry against *A. alternata* will help develop innovative and safer control strategies. Additionally, breeding and utilizing disease-resistant cultivars will increase their selectivity and reduce their dependence on chemical agents for disease management.

Plants have evolved to form complex and efficient protective mechanisms against the invasion of pathogenic bacteria, including cytoplasmic membrane phosphorylation, activation of cytoplasmic kinases, stimulation of reactive oxygen species (ROS) production, activation of Ca^2+^ channels, promotion of protein translation, and production of secondary metabolites, which ultimately result in immune responses in plants (Cohn et al., 2001; Schwessinger and Ronald, 2011). High-throughput sequencing technology is a next-generation sequencing technology widely used in plant disease research. Metabolomics is used to reflect the synthesis, decomposition, and transformation of all or certain types of metabolites in an organism’s tissue or cell (Hall, 2011; Khakimov et al., 2014). Transcriptomics and metabolomics have been used to study fungal interactions with non-model plant hosts, including soybean (Ranjan et al., 2019), jujube fruit (Yuan et al., 2019) and apple (Zhou et al., 2019). Biological stress is associated with reprogramming many defense-related genes and transcription factors. For example, citrus fruit inhibit *Penicillium digitatum* infection by enhancing ERF, WRKY, and MYB transcription, as well as the transcription of genes encoding stress, thus accumulating rhamnose and inositol (Ning et al., 2018). Gao et al. (2016) found that the response of peach trees to *Lasiodiplodia theobromae* infection was related to the expression of genes related to phenylpropanoid biosynthesis and glycometabolism. Abdelrahman et al. (2020) found that disease-resistant wild *Asparagus kiusianus* showed specific metabolic changes with increased flavonoids and steroidal saponins content. By contrast, limited information is available on the physiological and molecular response mechanism of cherry resistance to *A. alternata*, which would be the basis for successful disease resistance breeding. Therefore, the co-expression analysis of differential genes and metabolites *via* combined transcriptomic and metabolomic studies could be used to explore the causal relationship between genes and metabolites. Locking the key metabolic pathways and identifying candidate disease resistance genes may be an effective method to better explore and understand the mechanism of disease resistance in cherry to *A. alternata*.

The incidence and severity of leaf spot disease caused by *A. alternata* vary according to the cherry cultivar. We previously investigated different cherry cultivars for resistance to the natural incidence of the disease in the field and found that *P. cerasus* ‘Aode’ showed high resistance, while *P. avium* ‘Rita’ showed high sensitivity (data unpublished). Schuster and Tobutt evaluated the resistance of cherry genotypes to leaf spot disease and found tart cherry cultivars to be resistant and all tested sweet cherry cultivars to be susceptible (Schuster and Tobutt, 2004). Therefore, in the present study, we used the germplasm of cherry cultivars with marked differences in disease resistance as the research material to measure the physiological changes of cherry leaves during infection. Furthermore, we used transcriptome and metabolome sequencing to analyze the plant defense response pathways involving differentially expressed genes (DEGs) and differentially accumulated metabolites (DAMs), thereby revealing the potential defense mechanism of cherry against infection with *A. alternata*. The findings of this study could provide different perspective into how hosts fight the onslaught of fungal infections and accelerate the development and selection of resistant cherry cultivars.

## 2 Materials and methods

### 2.1 Plant materials and inoculation treatment

We selected *P. avium* ‘Rita’ (susceptible cherry cultivar (SC)) and *P. cerasus* ‘Aode’ (resistant cherry cultivar (RC)) as experimental materials. Among them, ‘Rita’ originated in Hungary and its parent is Trusenszkaja 2 × H2 (Germersdorfer open pollination) (Iličić et al., 2018), ‘Aode’ is a new sour cherry cultivar derived from wild sour cherry by seedling selection in China (Cai et al., 2014). One-year-old two selected cultivars grafted on *P. mahaleb* CDR-1 rootstocks were planted in the greenhouse at Northwest A&F University, Yangling, Shaanxi, China (34°20’ N, 108°24’ E). All test materials were maintained in the same growth state, grew robustly, and showed no pest infestation or diseases. The pathogen was isolated from diseased cherry leaves, and the isolate was confirmed to be *Alternaria alternata via* morphological culture characterization and molecular identification. *A. alternata* was incubated on potato dextrose agar (PDA) medium for 7 days at 25°C. The fourth and fifth open cherry leaves from the shoot tips were infected with a plaque of actively growing fungus (3 mm in diameter; excised using a punch to maintain consistency). Each leaf was inoculated with six pieces of fungus plaque (Zhu et al., 2017; Hu et al., 2018). Plant tissue was collected at the junction of diseased and healthy leaves using a clean straight-edged razor at 0, 1, 3, 5, 7, and 9 days post inoculation (dpi). The three biological replicates used for each treatment group.

### 2.2 Field emission scanning electron microscope observation of infected tissue

The morphology of cherry leaves inoculated with *A. alternata* was observed according to the method described by Liang et al. (Liang et al., 2020). The morphology of leaf tissue at 5 dpi was observed using FESEM (Hitachi, Tokyo, Japan). The cherry leaves were washed and cut into slices (length ≤ 5 mm, thickness ≤ 3 mm). Each slice was immersed and fixed in 4% (v/v) glutaraldehyde for more than 2 hours. Next, the slices were washed with 0.1 M PBS buffer (pH 6.8) four times, then washed with 30%, 50%, 70%, 80%, and 90% ethanol and washed three times with 100% ethanol. The ethanol was replaced with isoamyl acetate once. Finally, the tissue samples were dry gold plated and observed *via* FESEM.

### 2.3 Determination of the physiological index of cherry leaves

Different crude enzyme solutions from leaf tissue (0.2 g) were extracted using 5 mL of different extraction buffers. Sodium borate buffer (pH 8.8) containing 40 g/L polyvinylpyrrolidone (PVP) and 0.1mol/L acetate-sodium acetate buffer (pH 5.5) was used for PPO and POD extraction. Sodium phosphate buffer (0.1 mol/L; pH 7.8) was used for SOD and CAT extraction. The activities of PPO, POD, CAT, and SOD were determined according to our previously reported method (Pan et al., 2020; Pan et al., 2022). The activities were expressed as U/g based on fresh weight.

The MDA content was determined according to the method described by Dhindsa et al. (1981) with slight modifications. Cherry leaf samples (each 0.1 g) were homogenized in 5 mL of 5% (w/v) cold trichloroacetic acid solution, and centrifuged at 10,000 rpm for 10 min. Next, 2 mL of thiobarbituric acid was added to 2.0 mL of supernatant, and the reaction tubes were transferred to a boiling water bath for 20 min. The mixture was rapidly cooled and centrifuged again, and OD values were obtained at 420 nm, 532 nm, and 600 nm using a spectrophotometer.

The H_2_O_2_ content was determined using an assay kit (Beijing Soleibo Technology Co., Ltd., China) according to the manufacturer’s instructions. The H_2_O_2_ content was calculated on the basis of standard H_2_O_2_ at an absorbance of 415 nm and expressed as μmol•g ^-1^ FW.

The activities of CHI and GLU in cherry leaves inoculated with *A. alternata* were determined using the indicated kits (Beijing Solarbio Technology Co., Ltd.) according to the manufacturer’s instructions. Absorbance was measured at 585 nm and 540 nm using a full-wavelength multi-function microplate reader (Tecan Infinite M200pro, Switzerland). The result was expressed as U/g based on fresh weight.

The lignin content in cherry leaves inoculated with *A. alternata* at 0, 1, and 5 dpi was determined using the indicated kit (Beijing Solarbio Technology Co., Ltd.) according to the manufacturer’s instructions.

### 2.4 Transcriptome sequencing analysis

Cherry leaves at 0, 1, and 5 dpi after inoculation with *A. alternata* were used for RNA extraction, with three biological replicates per treatment. The raw data were obtained using the Illumina Novaseq6000 sequencing platform by Gene Denovo Biotechnology Co., Ltd. (Guangzhou, China). The paired-end clean reads were mapped to the cherry reference genome using HISAT2. 2.4 (Kim et al., 2015; Wang et al., 2020). Mapped reads for each sample were assembled using StringTie v1.3.1 (Pertea et al., 2015; Pertea et al., 2016), and RSEM was used to normalize gene transcription level to FPKM. We used DESeq2 (Love et al., 2014) to screen DEGs with false discovery rate (FDR) below 0.05 and |log_2_(fold change)| ≥2. DEGs were mapped to Gene Ontology (GO) and Kyoto Encyclopedia of Genes and Genomes (KEGG) databases to further analyze biological functions and key metabolic pathways.

### 2.5 Widely targeted metabolome analysis

Eighteen cherry leaf samples (each treatment included three biological replicates) were crushed using a mixer mill (MM 400, Retsch) at 30 Hz for 1.5 minutes. Next, 100 mg of cherry leaf powder was extracted with 70% aqueous methanol, and the extracted compounds were analyzed using an LC-ESI-MS/MS system (Chen et al., 2013). Data were filtered, detected peaks, aligned, and calculated using Analyzer 1.6.1. The DAMs between two groups were determined by P < 0.05 for the T-test and VIP ≥ 1. Next, a DAM pathway enrichment analysis was performed *via* the KEGG database.

### 2.6 Association analysis of transcriptome and metabolome data

The disease resistance responses of differential genes and differential metabolites of RC and SC were simultaneously mapped to the KEGG pathway database using GenMAPP v2.1 to obtain information about their common pathways.

### 2.7 RT-qPCR verification

RNA was reverse transcribed using the cDNA synthesis SuperMix kit (Yeasen Biotech Co., Ltd., Shanghai, China). RT-qPCR was performed using our previous method (Pan et al., 2022). Gene-specific primers were listed in **Table S9**. All reactions included three biological replicates. The relative gene expression level was calculated according to the 2^-△△^CT method (Livak and Schmittgen, 2002), with *P. avium* actin gene as an internal standard.

### 2.8 Plate inhibition assay of *A. alternata*

Growth inhibition assays of *A. alternata* were performed on culture plates using the growth rate method. PDA medium was prepared using 0 (blank control), 250, 500, or 1000 μg/mL of phenylalanine or tyrosine and DMSO (dimethyl sulfoxide) as the control solvent. PDA medium with DMSO was also used as a control to exclude the effect of DMSO on the experiment. The plates were inoculated with active *A. alternata* and grown at 25°C for 7 days to assess colony diameter. The assay was repeated three times for each concentration, and the inhibitory rate was calculated as follows: inhibitory rate = [(colony diameter of control – colony diameter of treatment)/colony diameter of control] × 100%.

### 2.9 Effect of coumarin on mycelial growth of *A. alternata* in cherry leaves

The 500 μg/mL coumarin solution was evenly sprayed on the surface of RC leaves, and then *A. alternata* was inoculated, the inoculation method is the same as described in Section 2.1. In order to improve the effect of coumarin, the leaves were moisturized with transparent preservative bags for 24 h. The lesion diameter was observed and measured at 5 dpi.

## 3 Results

### 3.1 Changes in symptoms following *A. alternata* infection

**Figure 1:**
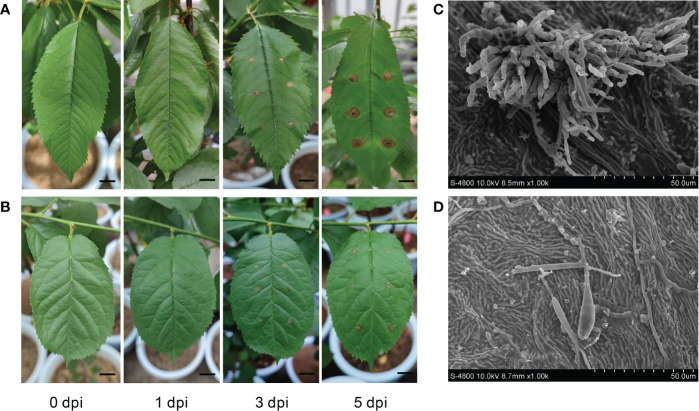
Analysis on symptoms and physiological indexes of *Alternaria alternata* infection in susceptible cultivar (SC) and resistant cultivar (RC). Disease symptoms of the **(A)** SC and **(B)** RC were observed following leaf inoculation with actively growing mycelia of *A. alternata* at 0, 1, 3, and 5 days post inoculation (dpi); scale bar = 10 mm. **(C)** Images showing infection in the SC at a magnification of ×1.00 k at 5 dpi. **(D)** Images showing infection in the RC at a magnification of ×1.00 k at 5 dpi.

*P. avium* ‘Rita’ (SC) and *P. cerasus* ‘Aode’ (RC) were selected based on our previous evaluation of different cherry cultivars for resistance to *A*. *alternata*. At 1 dpi with *A. alternata*, initial symptoms began appearing in the susceptible cultivar as light brown spots around the inoculation site that spread with the disease progressed, a darkening of the color was observed at the infection site at 5 dpi (**Figure 1A**). By contrast, lesion enlargement was limited on the leaves of the RC cultivar (**Figure 1B**). We measured the development of lesion diameter after inoculation with *A. alternata*, the lesion diameter of RC was only 63.14% of SC at 5 dpi and was significantly smaller than SC throughout the infection process (**Figure S1**). The inoculated leaves were observed using a FESEM, and the germination rate of conidia of the pathogen was higher on the leaf surface of the SC at 5 dpi. Moreover, a higher number of germ tubes—formed *via* spore germination—directly invaded the host tissue, and more mycelia entered the infected tissue *via* the stomata (**Figure 1C**). Conversely, the germination rate of conidia was significantly reduced in the RC leaf. Only scattered hyphae and single conidia were detected, and few germ tubes were produced (**Figure 1D**).

### 3.2 Physiological changes in cherry leaves inoculated with *A. alternata*

**Figure 2:**
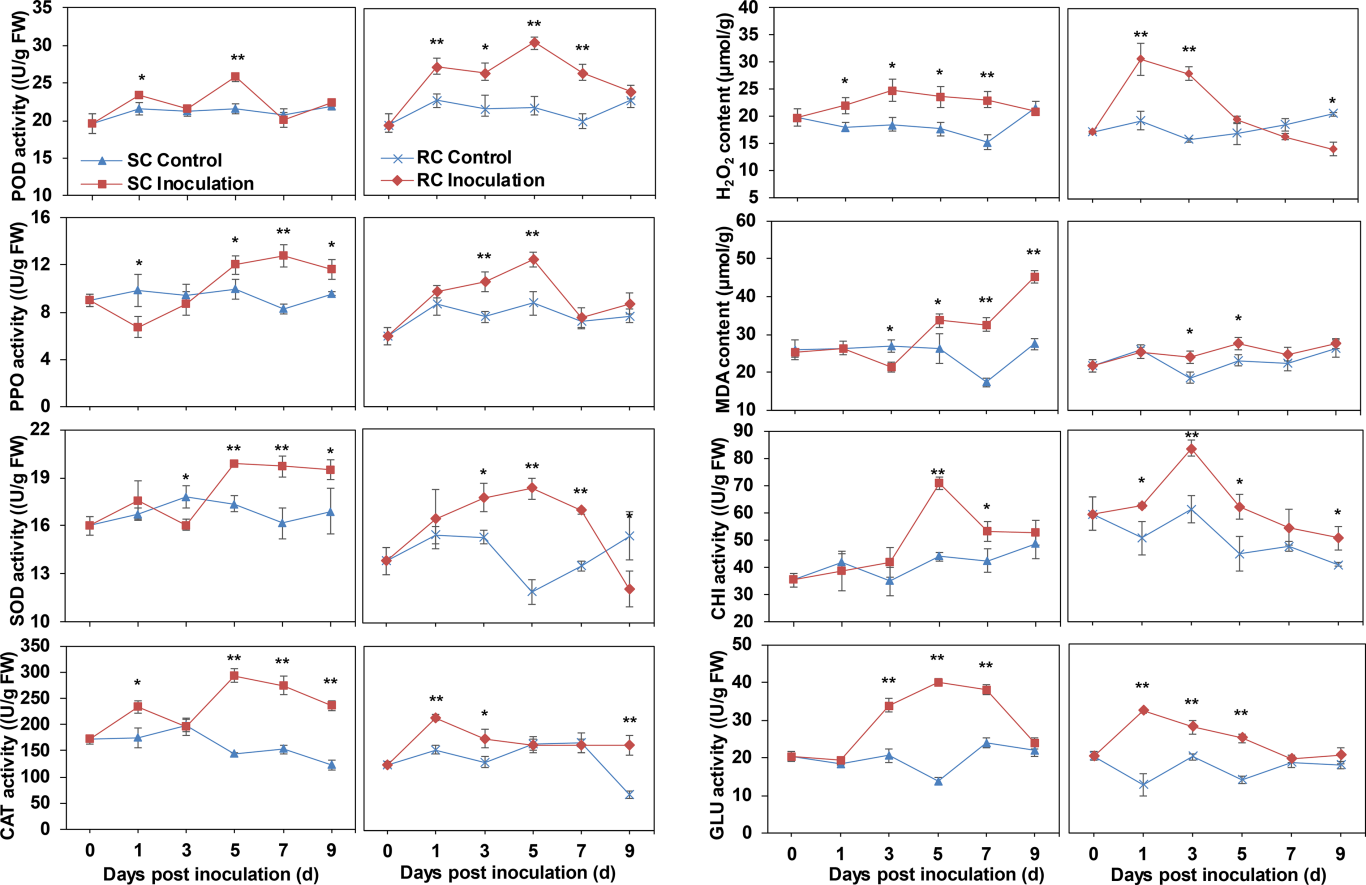
Determination of physiological indexes related to disease resistance, including peroxidase (POD), polyphenol oxidase (PPO), superoxide dismutase (SOD), catalase (CAT) enzyme activity, hydrogen peroxide (H_2_O_2_) and malondialdehyde (MDA) content, chitinase (CHI) and β-1,3 glucanase (GLU) enzyme activity. Error bars represent standard deviation (n = 3). * indicates a significant difference at p-value < 0.05, ** indicates a significant difference at p-value < 0.01.

Antioxidant enzymes can transform peroxides in the plant into less toxic or harmless substances. Here, peroxidase (POD), polyphenol oxidase (PPO), and superoxide dismutase (SOD) activities in the RC were significantly higher than those in the SC at the early stage of *A. alternata* inoculation, whereas the response of the SC to pathogenic fungi occurred after 5 dpi. Catalase (CAT) activity was significantly higher in the SC than in the uninoculated leaves (except at 3 dpi) and accumulated significantly in the RC at 1 dpi before leveling off. Following inoculation with *A. alternata*, the hydrogen peroxide (H_2_O_2_) content of the SC was significantly higher than that of the uninoculated group, while the H_2_O_2_ content of the RC was significantly higher than that of the uninoculated group at 1–3 dpi, suggesting that H_2_O_2_ accumulation was induced by pathogen infection (**Figure 2**).

The malondialdehyde (MDA) content of the SC showed an upward trend with the extension of infection time and increased sharply, especially after 5 dpi. By contrast, the content of MDA in the RC changed little during the infection process and had significantly difference with control only at 3, and 5 dpi (**Figure 2**).

In addition, the chitinase (CHI) and β-1,3 glucanase (GLU) activities were up-regulated after cherry leaves were infected by *A. alternata* (**Figure 2**). However, activity in the RC was significantly higher than in the uninoculated leaves during the early stage of inoculation but significantly higher in the SC than the control during the later stage of inoculation. Therefore, we speculated that resistance was induced and responded by the fungal pathogen earlier in the RC than the SC.

We considered that the resistance of cherry leaves to *A. alternata* may be more sensitive in the early stage of infection, based on data obtained on the physiological changes of cherry leaves following inoculation with *A. alternata*, in conjunction with leaf disease development. Therefore, cherry leaves were collected for transcriptome and metabolome determination at 0, 1, and 5 dpi.

### 3.3 Transcriptomic analysis of cherry in response to *A. alternata*

#### 3.3.1 Overview and analysis of transcriptome sequencing data

**Figure 3:**
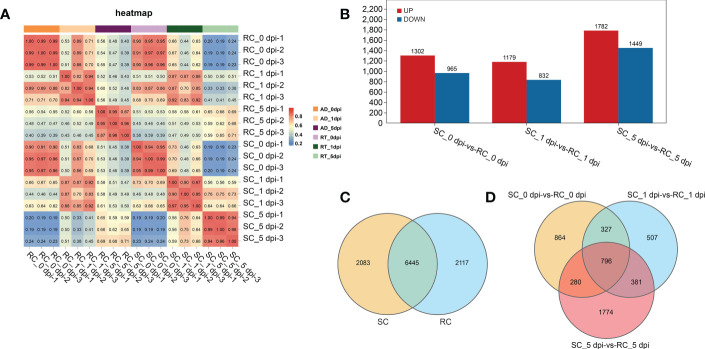
Analysis of the sample relationship and differentially expressed genes (DEGs) between groups. **(A)** Pearson correlation coefficients of all 18 samples; the correlation coefficients between the SC and RC were visually displayed as a heat map. **(B)** Up-regulation and down-regulation of DEGs. **(C)** A total of 2083 and 2117 DEGs were unique to the SC and RC, respectively. **(D)** DEGs in the SC compared to the RC at 0, 1, and 5 dpi.

RNA-seq data were generated from nine RC and nine SC samples at different stages (0, 1, and 5 dpi). A total of 931,889,502 clean reads were obtained after filtering and removing adapter sequences and low-quality reads, and the percentages of GC and Q20 were 45.28–46.31% and 98.06–98.32%, respectively (**Table S1**). Correlation matrix analysis showed that the biological duplication was satisfactory and suitable for subsequent analysis (**Figure 3A**).

#### 3.3.2 DEGs analysis of cherry in response to *A. alternata* infection

The gene expression level was estimated using the gene expression abundance corresponding to the FPKM value, and differential gene analysis was performed with P < 0.05 and |log_2_FC| ≥ 2 as the screening criteria. The sequencing results showed 10,645 DEGs in the RC and SC during the infection process. A pairwise comparison of DEGs was performed between the RC and SC at specific time points, and 2315 and 2053 DEGs were identified at 0 dpi and 1 dpi, respectively; the number of DEGs increased sharply after 5 dpi (**Figure 3B**). Among these, 6445 DEGs were differentially regulated in both the RC and SC, 2117 DEGs were only found in the RC, and 2083 DEGs were only found in the SC (**Figure 3C**). **Figure 3D** shows the overlapping genes between these groups. These results indicate that *A. alternata* infection resulted in notable changes in cherry gene expression and significant differences between the RC and SC.

We mapped DEGs to Gene Ontology terms (http://www.geneontology.org/) to find significantly enriched GO entries. GO analysis revealed that DEGs were classified into three ontologies, including biological processes (BPs), molecular functions (MFSs), and cellular components (CCSs) (**Figure S2**). Most DEGs (P ≤ 0.05) enriched in BPs were related to disease resistance, including the monoterpene metabolic process, phenylpropanoid metabolic process, phenylpropanoid biosynthetic process, jasmonic acid (JA) metabolic process, and hormone-mediated signaling pathway, at 1 and 5 dpi. MFs were mainly enriched in catalytic activity, oxidoreductase activity, and kinase activity, while CCs were mainly enriched in the extracellular region, cell periphery, external encapsulating structure, and cell wall (**Table S2**).

**Figure 4:**
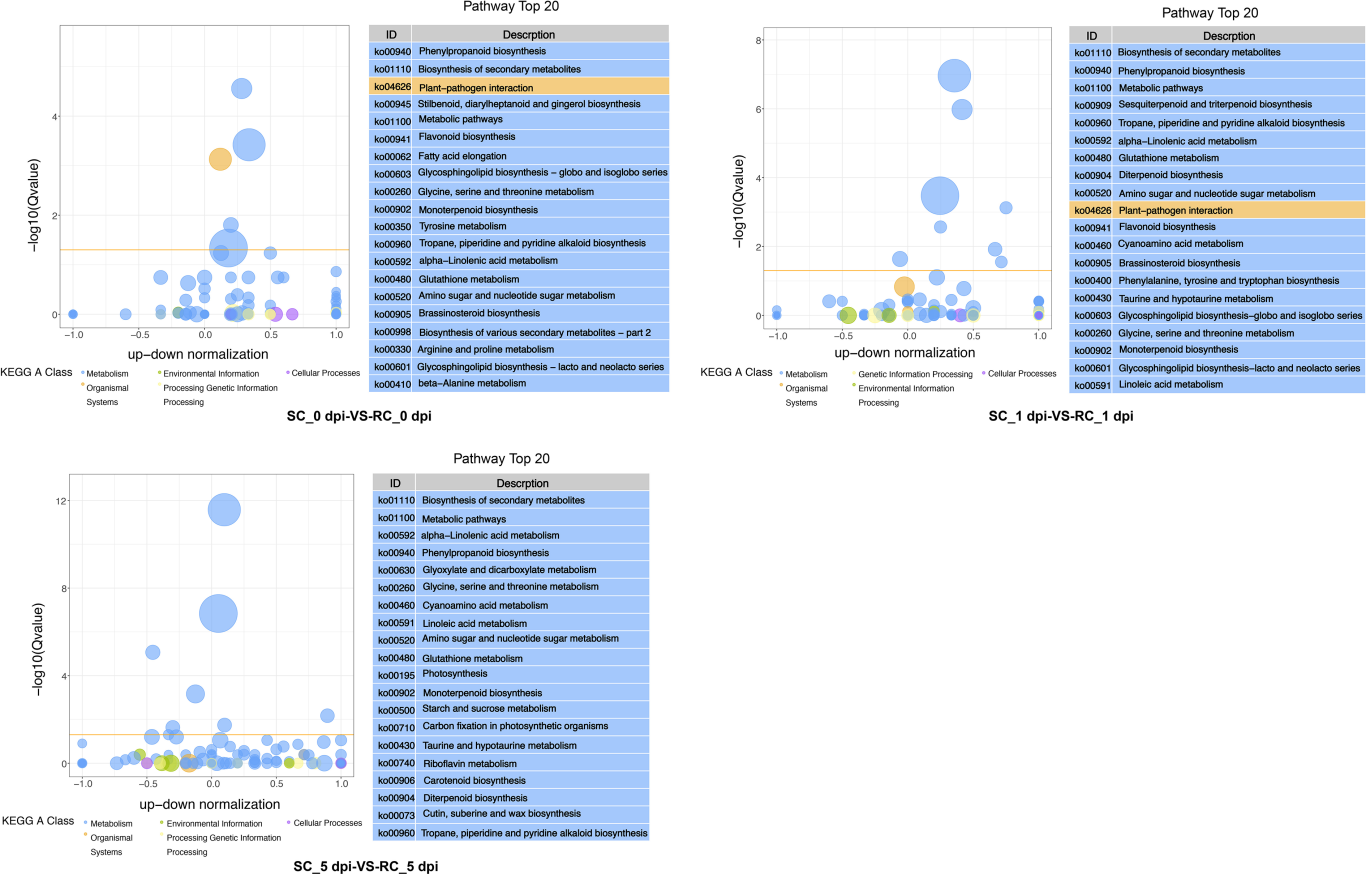
KEGG pathway analysis of pairwise comparisons (SC vs. RC) at 0, 1, and 5 dpi; the chart shows the top 20 pathways enriched in the selected group. The yellow line represents the threshold of Qvalue=0.05, and different colors represent different KEGG A class.

All DEGs were enriched to KEGG, and the enrichment pathways related to disease resistance were identified. DEGs between RC and SC were enriched in phenylpropanoid biosynthesis, α-linolenic acid metabolism, biosynthesis of secondary metabolites, tropane, piperidine and pyridine alkaloid biosynthesis, metabolite biosynthesis, glutathione metabolism, and amino sugar and nucleotide sugar metabolism in all three periods. Plant–pathogen interaction, flavonoid biosynthesis, and brassinosteroid biosynthesis were enriched at 0 and 1 dpi. Sesquiterpenoid and triterpenoid biosynthesis, phenylalanine, tyrosine and tryptophan biosynthesis were only enriched at 1dpi. DEGs were also enriched in photosynthesis, starch and sucrose metabolism, and cutin, suberin, and wax biosynthesis at 5 dpi (**Figure 4**). These results suggested that cherry has evolved various molecular defense reaction according to the infection stage of pathogenic fungi.

### 3.4 Metabolite profiling of cherry in response to *A. alternata*

#### 3.4.1 Quality control of metabolomic data

Metabolome analysis was performed to determine the condition of the metabolites in the RC and SC after the occurrence of disease resistance. The widely targeted metabolite profiling of extracted disease-resistant and susceptible cherry samples was carried out using an LC-ESI-MS/MS system. A total of 1088 metabolites were detected and classified into 35 categories, among which the top three were phenolic acids, flavonols, and amino acids and derivatives (**Table S3**). A heatmaps were constructed by normalizing metabolite content (**Figure S3A**) and principal component analysis (PCA) was subjected. PC1 and PC2 explained more than 52.4% of the variability (**Figure S3B**). We used partial least squares discriminant analysis to analyze multivariate metabolite data (**Figure S3C**). The analysis showed that different metabolomic characteristics manifested at each time point during the infection process and that the resistant and susceptible materials showed a relatively large separation.

#### 3.4.2 DAMs analysis of cherry in response to *A. alternata* infection

The DAMs between the two comparisons were screened by combining multivariate statistical analysis (OPLS-DA; VIP value) and univariate statistical analysis (T-test; P-value). The threshold for DAMs was VIP ≥ 1 and P < 0.05. The number of up- and down-regulated DAMs was 48 and 58, respectively, in SC_0 dpi-*vs*-RC_0 dpi, 45 and 69, respectively, in SC_1 dpi-*vs*-RC_1 dpi, and 58 and 78, respectively, in SC_5 dpi-*vs*-RC_5 dpi, which increased with the extension of inoculation time (**Figure S4**). The top 10 DAMs that increased or decreased in each comparison group were identified by changes in metabolite accumulation (**Figure S5**).

**Figure 5:**
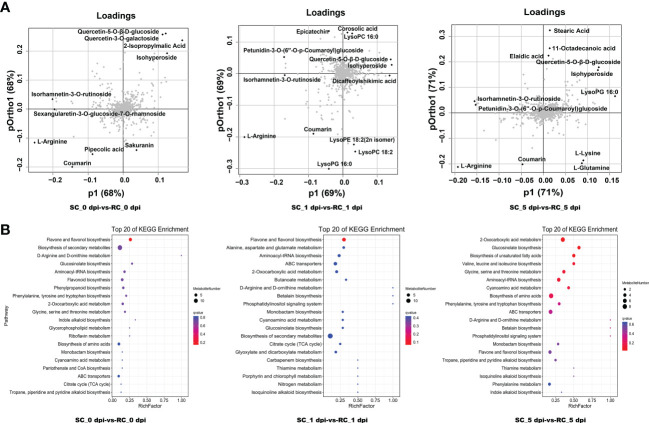
Analysis of differentially accumulated metabolites (DAMs) detected between the SC and RC. **(A)** Loading plots of DAMs. **(B)** KEGG pathway analysis of DAMs, the chart shows the top 20 pathways enriched in the selected group.

Loading plots help to identify the metabolites that contribute the most to the changes in metabolite patterns between the comparison groups, i.e., the variables that contribute the most to the principal components of the OPLS model. Our findings showed that most DAMs far from the origin belonged to flavonoids and lipid metabolites (**Figure 5A**). The metabolites petunidin-3-O-(6’’-O-p-Coumaroyl) glucoside, isorhamnetin-3-O-rutinoside, quercetin-5-O-β-D-glucoside, isohyperoside, L-Arginine, and coumarin were all far from the origin and contributed more to the discrimination between the two sample groups at the three time points. These are likely to be important variables leading to sample separation.

#### 3.4.3 KEGG enrichment analysis of DAMs

Based on the KEGG database annotations, we listed the top 20 paths enriched in each comparison group (**Figure 5B**). Among them, DAMs were enriched in flavone and flavonol biosynthesis, ABC transporters, flavonoid biosynthesis, cyanoamino acid metabolism, tropane, piperidine and pyridine alkaloid biosynthesis, and phenylpropanoid biosynthesis, following inoculation with *A. alternata*. These metabolic pathways responded positively to the defense response of cherry. The results of enrichment analysis were consistent with those of transcriptome analysis. We noticed that only the metabolites kaempferol-3-O-galactoside and kaempferol-3-O-glucoside from flavone and flavonol biosynthesis were significantly up-regulated in the RC at all three stages, whereas the other flavonoid metabolites accumulated in the SC, which may suggest a specific response of the RC to *A. alternata* (**Table S4**).

### 3.5 Association analysis of transcriptome and metabolome data

#### 3.5.1 Common metabolic pathway between genes and metabolites

We performed a combined transcriptome and metabolome analysis to understand further the mechanism of cherry resistance to *A. alternata* infection. KEGG enrichment analysis showed that comparisons of SC_0 dpi-*vs*-RC_0 dpi, SC_1 dpi-*vs*-RC_1 dpi, and SC_5 dpi-*vs*-RC_5 dpi had 31, 47, and 42 metabolic pathways co-mapped, respectively (**Table S5**). The main enriched metabolic pathways included phenylpropanoid biosynthesis, α-linolenic acid metabolism, flavonoid biosynthesis, biosynthesis of secondary metabolites, tropane, piperidine, and pyridine alkaloid biosynthesis, glutathione metabolism, ABC transporters, cyanoamino acid metabolism, and aminoacyl-tRNA biosynthesis. Considering that transcriptome analysis highlighted the important role of α-linolenic acid metabolism and phenylpropanoid biosynthesis in plant defense, we focused on these two metabolic pathways in subsequent analyses.

#### 3.5.2 JA signaling in response to resistance against *A. alternata*

**Figure 6:**
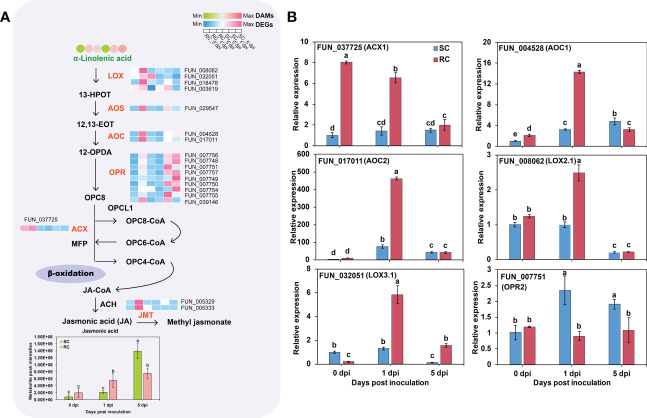
Jasmonic acid (JA) synthesis pathway and gene expression analysis. **(A)** Schematic representation of the core JA synthesis pathway; the circle heat map and the square heat map represent the expression of DAMs and DEGs, respectively, in the SC and RC at the three stages of testing. **(B)** RT-qPCR analysis of JA synthesis-related genes. Error bars represent standard deviation (n = 3). Different letters above the bars indicate significant differences at the 0.05 level according to Duncan’s multiple range test.

GO analysis indicated that DEGs between the RC and SC were significantly enriched in JA metabolism in biological processes at the transcript level (**Table S2**). KEGG analysis highlighted α-linolenic acid metabolic pathways associated with JA synthesis and response. The expression levels of three genes (FUN_008062, FUN_032051, and FUN_018478) encoding lipoxygenase (LOX) were up-regulated in the α-linolenic acid metabolic pathway. Two genes (FUN_017011 and FUN_004528) that encode allene oxide cyclase (AOC) were up-regulated in the RC during the early stage of *A. alternata* inoculation but down-regulated at 5 dpi, compared with the SC. In addition, the expression levels of one gene (FUN_029547) encoding hydroperoxide dehydratase (AOS) and nine genes encoding 12-oxophytodienoic acid reductase (OPR) were all down-regulated at 5 dpi (**Figure 6A**, **Table S6**). The RNA-seq data was validated *via* RT-qPCR (**Figure 6B**). Furthermore, JA content was significantly accumulated in the RC at 0 and 5 dpi. In addition, the accumulation of linolenic acid, a precursor of JA, was significantly less in the RC than in the SC at 5 dpi. Therefore, the differences in defense responses may be caused by different stages of *A. alternata* infection, and the sharp increase of key genes involved in JA synthesis at later stages in the SC may be considered a delayed response to the fungus.

#### 3.5.3 The phenylpropanoid pathway in response to resistance against *A. alternata*

**Figure 7:**
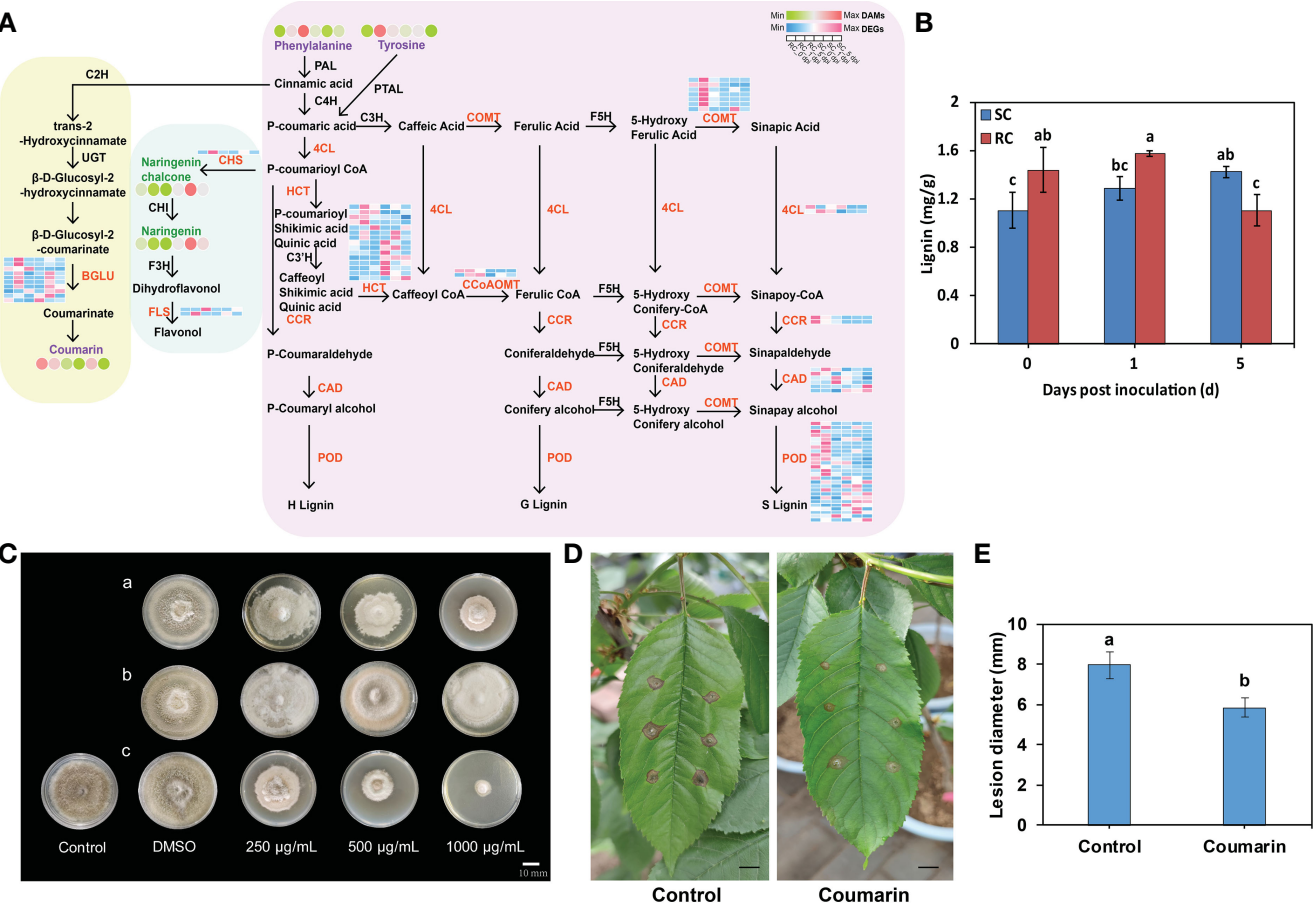
DEGs and DAMs involved in the phenylpropanoid pathway in the SC and RC inoculated with *A alternata*. **(A)** Schematic representation of the core pathway of phenylpropanoid biosynthesis; the circle heat map and the square heat map represent the expression of DAMs and DEGs, respectively, in the SC and RC at the three stages of testing. **(B)** The lignin content of the SC and RC at 0, 1, and 5 dpi. Error bars represent standard deviation (n = 3). Different letters above the bars indicate significant differences at the 0.05 level according to Duncan’s multiple range test. **(C)** Effect of the metabolites on *A. alternata* growth. Tyrosine (a) and coumarin (c) inhibit the growth of *A. alternata*, while phenylalanine (b) does not. DMSO (dimethyl sulfoxide) was used as the solvent control. Scale bar = 10 mm. **(D)** Effect of coumarin on growth of *A. alternata* in RC leaves. Scale bar = 10 mm. **(E)** Effect of coumarin on lesion diameter of RC leaves. Error bars represent standard deviation (n = 3). Different letters above the bars indicate significant differences at the 0.05 level according to Duncan’s multiple range test.

Plants have evolved various branched pathways for phenylpropanoid metabolism, producing numerous metabolites such as flavonoids, lignin, lignans, and cinnamic acid amides (Dong and Lin, 2021). Here, DEGs and DAMs related to the phenylpropanoid pathway were noticed between the RC and SC. We observed an up-regulation of genes encoding cinnamyl-alcohol dehydrogenase (CAD), peroxidase (POD), caffeoyl-CoA O-methyltransferase (CCoAOMT), 4-coumarate-CoA ligase (4CL), cinnamoyl-CoA reductase (CCR), caffeic acid 3-O-methyltransferase (COMT), and scopoletin glucosyltransferase (TOGT) at the transcript level in RC compared to SC, as well as a down-regulation of genes encoding β-glucosidase (β-GLU) and shikimate O-hydroxycinnamoyltransferase (HCT) (**Figure 7A**, **Table S7**). Furthermore, the lignin content of RC was significantly higher than that of SC at the early stage of inoculation (**Figure 7B**). Moreover, the gene expression of chalcone synthase (CHS) and flavonol synthase (FLS) related to flavonoid biosynthesis was significantly up-regulated in the RC compared to SC, following pathogen induction. Naringin and naringin chalcone also showed significant differences (**Figure 6A**). We selected candidate genes for RT-qPCR to validate the RNA-seq data, both sets of results were consistent (**Figures S6A, B**).

Based on the metabolomic data, we found that the metabolites phenylalanine, tyrosine, and coumarin were significantly accumulated in the RC compared with the SC (**Figure 7A**). Subsequently, plate inhibition assays of *A. alternata* showed that increasing concentrations of tyrosine and coumarin significantly inhibited the growth of *A. alternata in vitro*, while phenylalanine had no significant inhibitory effect on *A. alternata* (**Figure 7C**, **Figure S7A**). The inhibition rates of coumarin and tyrosine on the mycelial growth of *A. alternata* were significantly higher than that of phenylalanine, and the inhibition rates were 86.02% and 53.68% at the mass concentration of 1000 μg/mL, respectively (**Figure S7B**). Among them, coumarin had the most significant inhibitory effect on *A. alternata*, and we hypothesized that coumarin may inhibit the growth of diseased spots on cherry leaves. We then verified the effect of coumarin on mycelial growth of *A. alternata* in cherry leaves. The results showed that coumarin significantly inhibited the growth of diseased spots on RC leaves (**Figures 7D, E**).

### 3.6 Transcription factors

**Figure 8:**
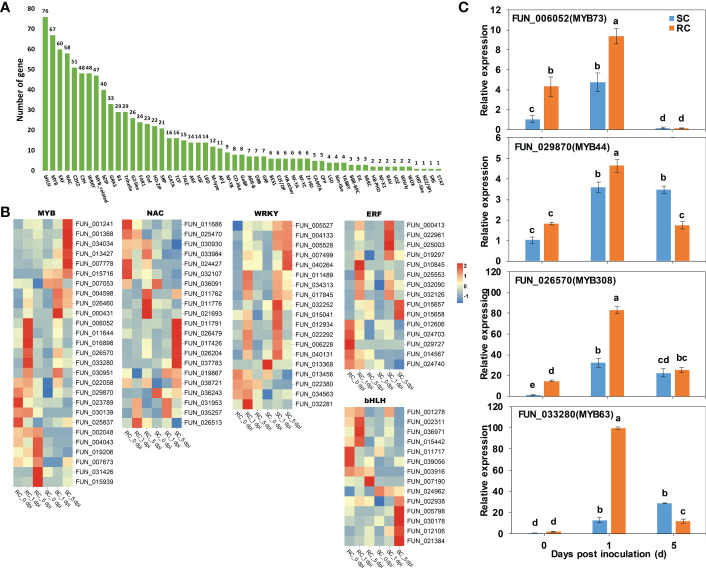
Analysis of transcription factors (TFs). **(A)** Number of TFs in SC and RC inoculated with *A. alternata*. **(B)** Heatmaps of DEGs encoding TFs, including MYBs, NACs, WRKYs, ERFs, and bHLHs. **(C)** RT-qPCR analysis to four MYB TFs involved in phenylpropanoid biosynthesis. Error bars represent standard deviation (n = 3). Different letters above the bars indicate significant differences at the 0.05 level according to Duncan’s multiple range test.

TFs play an important regulatory role in plant growth and development and defense responses to stress. Our RNA-seq analysis revealed that were 943 TFs, and the top five TFs included members of the WRKY, MYB, bHLH, NAC, and ERF families (**Figure 8A**). We compared the detailed trends of expression of the DEGs that encode these five TF families between the RC and SC based on significant levels of expression (P ≤ 0.05) (**Figure 8B**). A total of 27 genes in the MYB family differed significantly. Most of the MYB genes were significantly up-regulated in the RC (particularly at 0 and 1 dpi). We found that the gene expression levels of 10 NAC families were significantly up-regulated in the RC compared to the SC. Interestingly, most of the WRKY TFs responses manifested in RC inoculated with *A. alternata* at 0 and 1 dpi, whereas the SC responded to *A. alternata* infection at 1 dpi and 5 dpi. The bHLH and ERF families of TFs also positively responded to *A. alternata* infestation in the RC at the early stage of inoculation.

MYB TFs are involved in many metabolic pathways, and they also play an important role in the phenylpropanoid biosynthetic pathway (Albert et al., 2015; Guo et al., 2017). Combined with the results of a GO analysis (**Table S8**), four MYB TFs that were involved in phenylpropanoid biosynthesis were noted, and an RT-qPCR analysis showed that they were significantly up-regulated in the infection process. We found the level of expression of *PcMYB63* significantly increased at 1 dpi in the RC, which was 7.5-fold higher than that in the SC (**Figure 8C**).

## 4 Discussion

The determinants of *A. alternata* infestation in cherry and the subsequent complex network of responses remain poorly understood. Exploring the key components of these defense responses is critical to producing disease-resistant crops. In the present study, we used transcriptomic and metabolomic approaches to compare the response of two widely different cherry cultivars inoculated with *A. alternata* and explored sensing, signaling, and defense responses in the disease-resistance system of cherry. These results could further our understanding of the mechanism underlying resistance to *A. alternata* infection in cherry.

Plants under biotic or abiotic stress produce ROS, one of the earliest cellular responses after the host successfully recognizes the pathogen (Sagi and Fluhr, 2006). The balance between ROS synthesis and scavenging can ensure the basal metabolism of plant organisms during the process of stress response (Laloi et al., 2007). Here, the H_2_O_2_ content of cherry leaves infected with *A. alternata* was significantly higher than that of the uninoculated group. Moreover, the H_2_O_2_ content of the RC was higher than that of the SC at the early stage of pathogen infection. Therefore, the role of H_2_O_2_ in the RC may be to induce allergic necrosis of host cells, thereby inhibiting the spread of pathogens. The same phenomenon was observed in the interaction between wheat and stripe rust, with ROS found only in resistant cultivars (Wang et al., 2010). SOD can scavenge superoxide free radicals (O_2_^-^); it cooperates with CAT, POD, and other enzymes to defend against the damage of ROS or other peroxide free radicals to the cell membrane system (Elkomy, 2014; Pan et al., 2020). The activity levels of CAT, SOD, and POD remained high at the later stage of inoculation with *A. alternata*, perhaps to stabilize the intracellular H_2_O_2_ content. MDA is one of the major products of membrane lipid peroxidation, and its accumulation can damage plant cytoplasmic membranes and organelles (Cervilla et al., 2007), as evidenced by significant increases in the SC. CHI and GLU can directly degrade fungal cell walls and inhibit the growth of pathogenic bacteria (Mauch et al., 1988). Further, the degraded fungal cell walls can act as signaling substances to trigger the PAMP-triggered immunity (PTI) defense response in plants (Lawrence et al., 2000). The resistant *Cuminum cyminum* plant showed higher CHI and GLU activity against *Fusarium oxysporum* (Ravi et al., 2021), as did cherry against *A. alternata*. In general, the RC initiated resistance responses earlier and to a higher degree than the SC.

Combined transcriptome and metabolome analysis indicated that phenylpropanoid biosynthesis and JA biosynthesis in our cherry cultivars was regulated to varying degrees in response to *A. alternata* infection. JA, salicylic acid (SA), and ethylene (ET) are the three key hormones in plants that respond to the invasion of pathogens (Panstruga et al., 2009). The contents of SA, JA, and ET will establish a new balance after the plants perceive the invasion of pathogens; this precise regulation depends on the plant’s identification and judgment of pathogen types (Bari and Jones, 2009). SA is believed to primarily mediate plant disease resistance to biotrophic and hemibiotrophic pathogens, while JA and ET mainly mediate resistance against necrotrophic pathogens (Glazebrook, 2005). *A. alternata* is a typical necrotrophic pathogen. GO analysis of the transcriptome showed that the JA metabolic process was enriched in both SC_1 dpi-*vs*-RC_1 dpi and SC_5 dpi-*vs*-RC_5 dpi and that the SA-mediated signaling pathway was enriched both at 0 dpi and 5 dpi (**Table S2**). We speculated that SA might also promote the local immunity of cherry against the necrotrophic pathogen *A. alternata*. Similarly, SA and JA resistance pathways were induced in wheat and apple following inoculation with *F. graminearum* and *A. alternata*, respectively (Ding et al., 2011; Zhu et al., 2017). Previous studies have shown that JA mutants displayed increased resistance to certain necrotrophic pathogens. For example, JA biosynthesis mutant OPR3 showed super resistance to *F. graminearum*, and JA signal mutant MYC2/JIN1 showed enhanced resistance to *B. cinerea* and *P. cucumerina* (Bhattarai et al., 2008; Kachroo and Kachroo, 2009). Interestingly, our findings showed that most genes involved in JA biosynthesis, including LOX, AOC, AOS, and OPR, were significantly up-regulated in the RC at 1 dpi. By contrast, the expression of genes involved in JA biosynthesis remained low early in the SC but increased dramatically at 5 dpi, which could be considered a defense against established delayed or unsuccessful responses to pathogenic infection. Our metabolomic analysis also showed that the overaccumulation of linolenic acid occurred at 5 dpi in the SC, consistent with the expression pattern of the transcriptome. Similar results were obtained when investigating soybean resistance against *S. sclerotiorum* (Ranjan et al., 2019). Although JA signaling was activated in both the RC and SC, the resistance response of RC to *A. alternata* was consistent with the early JA signal in the interaction process, we speculate that the time of induction is essential for the outcome of resistance.

The phenylpropanoid pathway was reprogrammed, and phenylalanine, tyrosine, and coumarin accumulated significantly in the RC, with coumarin showing obvious inhibitory effects on the growth of *A. alternata* (**Figures 7C, D**). The coumarin synthesis pathway is a branch of the phenylpropanoid metabolic pathway, and the metabolite coumarin can resist the growth and reproduction of various plant pathogens, thus enhancing plant disease resistance (Kai et al., 2006). The coumarin derivative (5’-hydroxy-aurapten) extracted from *Lotus lalambensis* could inhibit conidial germination of *Aspergillus flavus* at concentration 40 µg/mL (Ali et al., 2021). Gao et al. (2019) found that coumarin can be used as an inducer of plant immune elicitor to induce ROS production and promoted rice blast resistance activity. It has also been reported that the antifungal effect of coumarin compounds is caused by the change of cell membrane permeability (Ayine-Tora et al., 2016). Therefore, coumarin is expected to be an effective natural antifungal agent for cherry. The lignin and flavonoid pathways are two other important branches of the phenylpropanoid biosynthesis pathway. Although phenylalanine had no significant inhibitory effect on *A. alternata* as an upstream metabolite of phenylpropanoid biosynthesis, it appeared to induce changes in downstream genes. We found that genes encoding CAD, POD, CCoAOMT, 4CL, CCR, and COMT were up-regulated, affecting the lignin content and flavonoid pathways, it was suggested that they may play a potential role in the resistance of cherry to *A. alternata*. A similar interaction was observed between rust-resistant maize strain and *Puccinia sorghi* (Kim et al., 2021). Because lignin biosynthesis is associated with cell wall strengthening as a disease-resistance mechanism, and it has a strong ability to scavenge ROS, the free radicals produced during lignin metabolism can simultaneously inactivate fungal cells (Tronchet et al., 2010; Zheng et al., 2021). Our findings supported this finding. Electron microscopy showed that only sporadic hyphae and single conidia were found on the leaves of the RC (**Figure 1D**). The lignin content of the RC was significantly higher than that of the SC in the early stage of inoculation (**Figure 7B**), and the genes involved in cell wall synthesis were highly expressed in the RC at 0 and 1 dpi, potentially explaining why *A. alternata* cannot invade the RC. The main physiological functions of flavonoids are their capacity to act as free radical scavengers and antioxidants. The up-regulation of CHS and FLS, as well as flavonoid accumulation, played a positive role in the resistance of cherry to *A. alternata*. The ROS scavenging capacity of flavonoids has been proposed to protect against pathogens, including *Sclerotinia sclerotiorum* (Ranjan et al., 2019) and *Phytophthora nicotianae* (Sun et al., 2022). These mechanisms may confer resistance against *A. alternata* in cherry.

MYB, NAC, WRKY, ERF, and bHLH are all important regulators of defense responses in plants (Ambawat et al., 2013; Zhu et al., 2017; Yuan et al., 2021). The MYB TFs in some plants enhance the plants defense against diseases by regulating the synthesis of phytoalexins. *VqMYB154* can regulate the accumulation of resveratrol to enhance the resistance of grapevine to pathogens (Jiang et al., 2021). Overexpression of *SlMYB75* regulated JA accumulation in tomato and promoted JA-mediated signaling against *B. cinerea* infection (Liu et al., 2020). Overexpression of grapevine *VvNAC1* in *Arabidopsis* improved resistance to *B. cinerea* and *Hyaloperonospora arabidopsidis* by regulating defense genes (Le Hénanff et al., 2013). Overexpression of *VaWRKY10* in *Arabidopsis thaliana* and *Vitis vinifera* Thompson Seedless enhanced resistance to *B. cinerea* (Wan et al., 2021). Transgenic rice plants overexpressing *OsERF83* showed substantial inhibition of lesion formation following rice blast infection (Tezuka et al., 2018). Overexpression of *OsbHLH057* enhanced disease resistance and drought tolerance in rice (Liu et al., 2022). Our results showed that specific TFs were significantly higher expression in the RC than in the SC. In particular, we found four MYB TFs that were involved in phenylpropanoid biosynthesis were significantly up-regulated. We hypothesized that these TFs may have important regulatory and transport roles in cherry during resistance against *A. alternata* infection. The role of TFs in the disease resistance of sweet cherry has not been thoroughly investigated, future work will focus on the in-depth analysis of these TFs to determine their functions.

## 5 Conclusion

**Figure 9:**
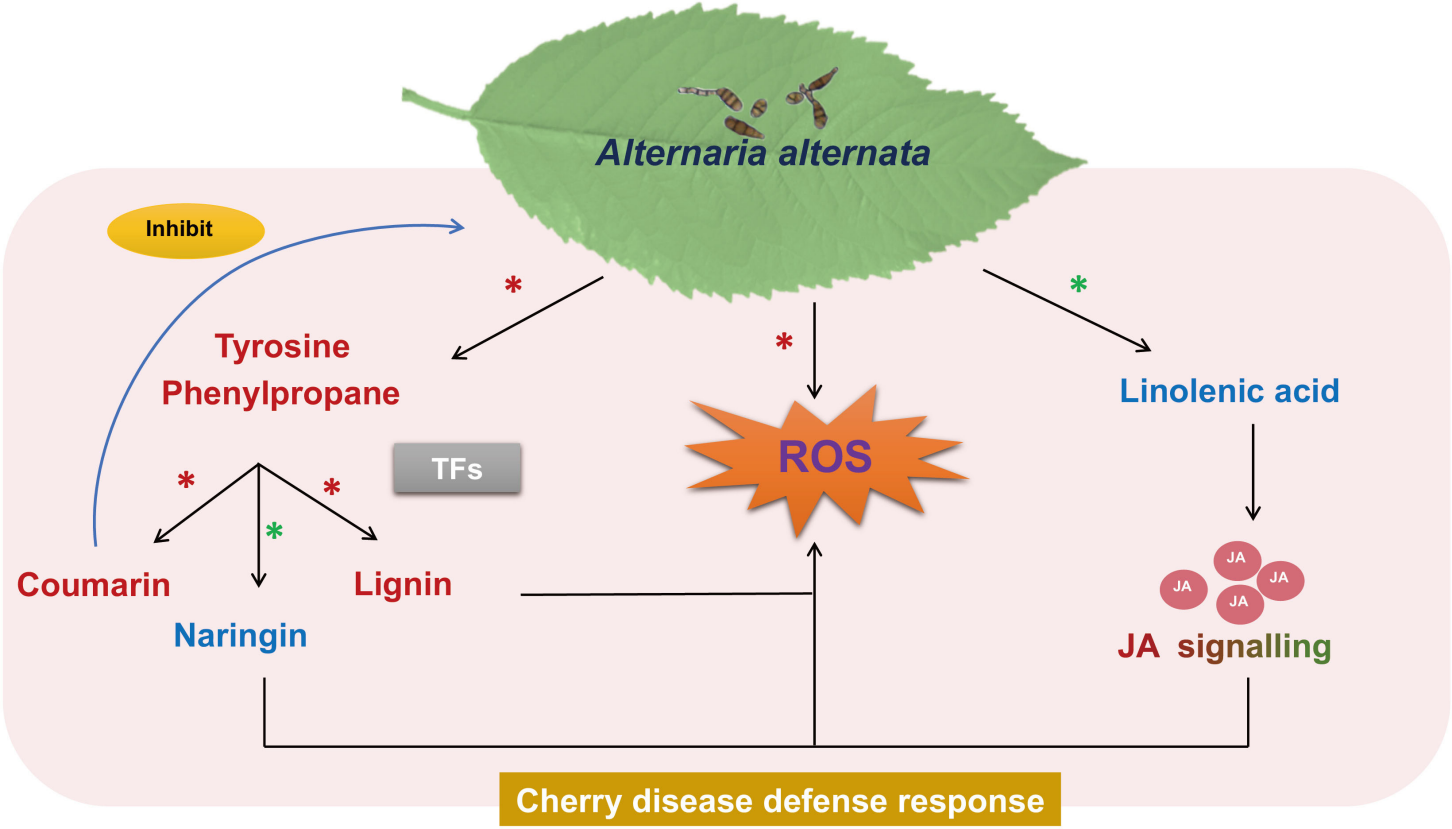
The proposed defense response mechanism of cherry to *A. alternata* infection. ROS are the core defense responses of cherry to *A. alternata*; JA, phenylpropanoid, and flavonoid biosynthesis are the key pathways, coumarin could effectively inhibit the growth of *A. alternata*, and TFs could be the key responsive factor in the response of cherry to *A. alternata*. The black line is induction of a process, blue line represents inhibition. Metabolite name indicated in red or blue represent significant accumulation in the RC or SC, respectively. The words indicated by gradient colors, which represent the significant accumulation of JA signaling in RC at an early stage. Red asterisks (*) indicate up-regulation in the resistance response, green asterisks (*) indicate down-regulation in the resistance response.

In this study, we proposed a mechanism of the defense response of cherry to *A. alternata* infection (**Figure 9**). The resistant cultivar exhibited higher antioxidant abilities and stronger cell wall defense at the physiological levels. Transcriptomic and metabolomic analyses revealed the activation of secondary metabolic pathways, including JA, phenylpropanoid, and flavonoid biosynthesis, during the defense responses of cherry against *A. alternata*. The resistance of cherry to *A. alternata* was associated with the early accumulation of JA. Notably, since coumarin could effectively inhibit the growth of *A. alternata*, it is expected to become an available natural antifungal agent for cherry. Moreover, several TFs (e.g., MYB, NAC, WRKY, ERF, and bHLH) that may be involved in *A. alternata* -mediated defense responses by activating disease resistance signaling and downstream defense pathways. Our study provides deeper insights into understanding the mechanisms of resistance of cherry against infection by *A. alternata*.

## Data availability statement

The datasets presented in this study can be found in online repositories. The names of the repository/repositories and accession number(s) can be found below: https://www.ncbi.nlm.nih.gov/, PRJNA893261.

## Author contributions

L-YP, Y-LC and TW conceived and designed the research; L-YP, JiZ, YS, B-XQ, and R-QG performed the experiments; L-YP, JuZ, and D-QS analyzed the results; L-YP wrote the manuscript; and Y-LC and TW revised the manuscript. All authors contributed to the article and approved the submitted version.
