# Supplementary material for: Comparative transcriptome and metabolome analyses of cherry leaves spot disease caused by Alternaria alternata
Source: Front Plant Sci. 2023 Feb 9;14:1129515. doi: 10.3389/fpls.2023.1129515 (PMC9947566; doi:10.3389/fpls.2023.1129515)
Supplement: Supplementary file 1 [file DataSheet_1.docx]

Supplementary Material

Comparative Transcriptome and Metabolome Analyses of Cherry Leaves Spot Disease Caused by *Alternaria alternata*

Liu-Yi Pan^1^, Jing Zhou^1^, Yan Sun^1^, Bai-Xue Qiao^1^, Tian Wan^1^, Rui-Quan Guo^1^, Juan Zhang^1, 2^, Dong-Qian Shan^1^, Yu-Liang Cai^1*^

^1^ College of Horticulture, Northwest A&F University, Yangling 712100, Shaanxi, China.

^2^ College of Horticulture and Forestry, Tarim University, Alar 843300, Xinjiang, China.

*** Correspondence:**Yu-Liang Cai
[yuanyicyl@nwsuaf.edu.cn](mailto:yuanyicyl@nwsuaf.edu.cn)

# Supplementary Tables

**Table S1** Summary of the sequencing data generated on the Illumina platform

**Table S2** The enrichment degree of the top twenty GO pathways of differentially expressed genes (DEGs)

**Table S3** Results of metabolite identification

**Table S4** Differentially accumulated metabolites (DAMs) in the flavone and flavonol biosynthesis pathways

**Table S5** KEGG enrichment analysis of DEGs and DAMs enriched in the same pathway

**Table S6** A list of DEGs within the cherry alpha-linolenic acid metabolism

**Table S7** A list of DEGs within the cherry phenylpropanoid biosynthesis

**Table S8** GO enrichment analysis of MYB transcription factors

**Table S9** Primers used in this study

# Supplementary Figures

**Figure S1** Development of leaves lesion diameter after inoculation with *Alternaria alternata***.** Error bars represent standard deviation (n = 3). Different letters above the bars indicate significant differences at the 0.05 level according to Duncan’s multiple range test.


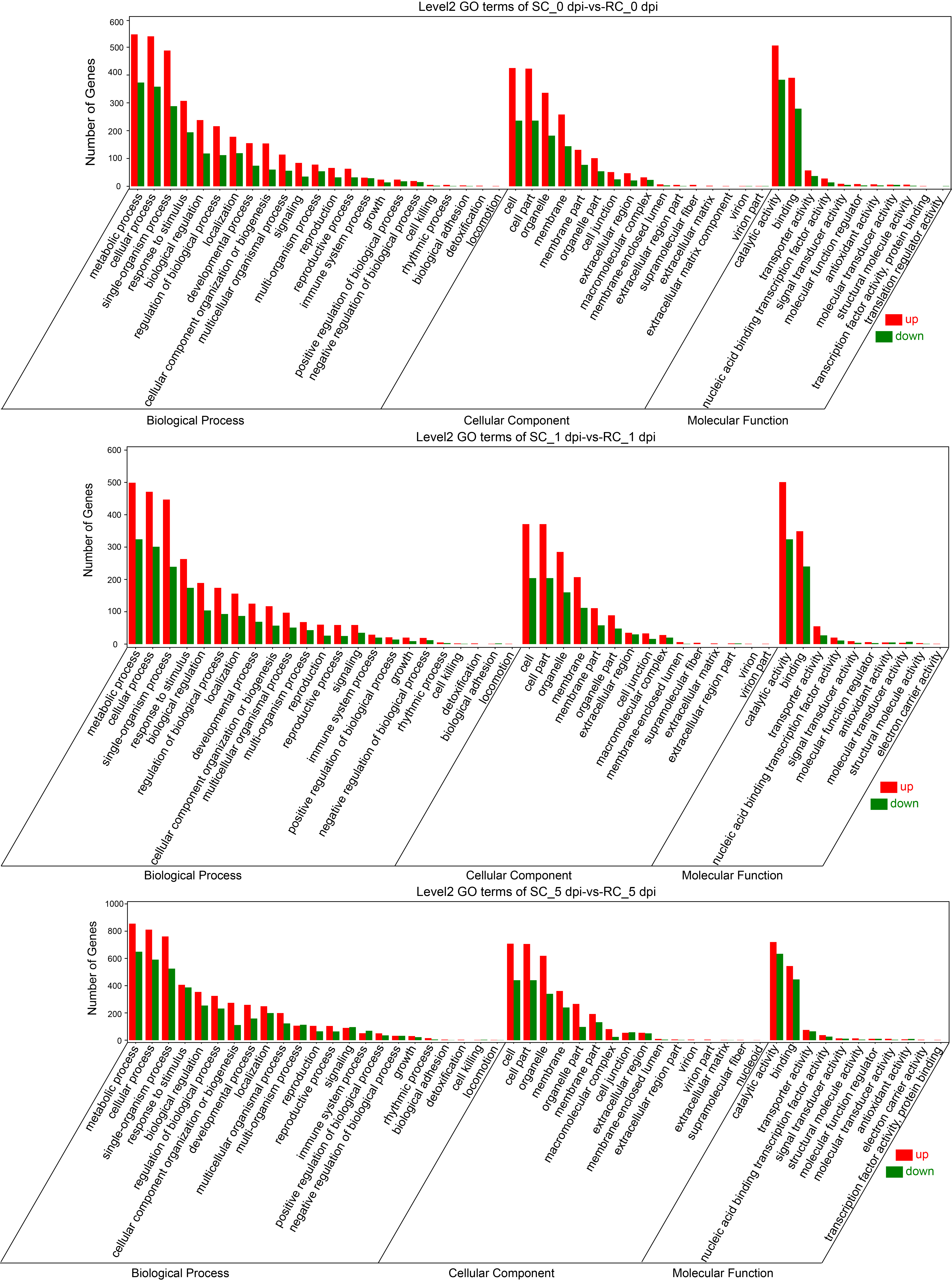


**Figure S2** Gene ontology (GO) functional enrichment in the resistant cultivar (RC) compared to the susceptible cultivar (SC) at different time points following infection with *A. alternata*. The y-axis indicates the total number of genes annotated to each GO process. The y-axis represents three ontologies, including biological processes, molecular functions, and cellular components. The red and green sections represent up-regulated and down-regulated genes, respectively.


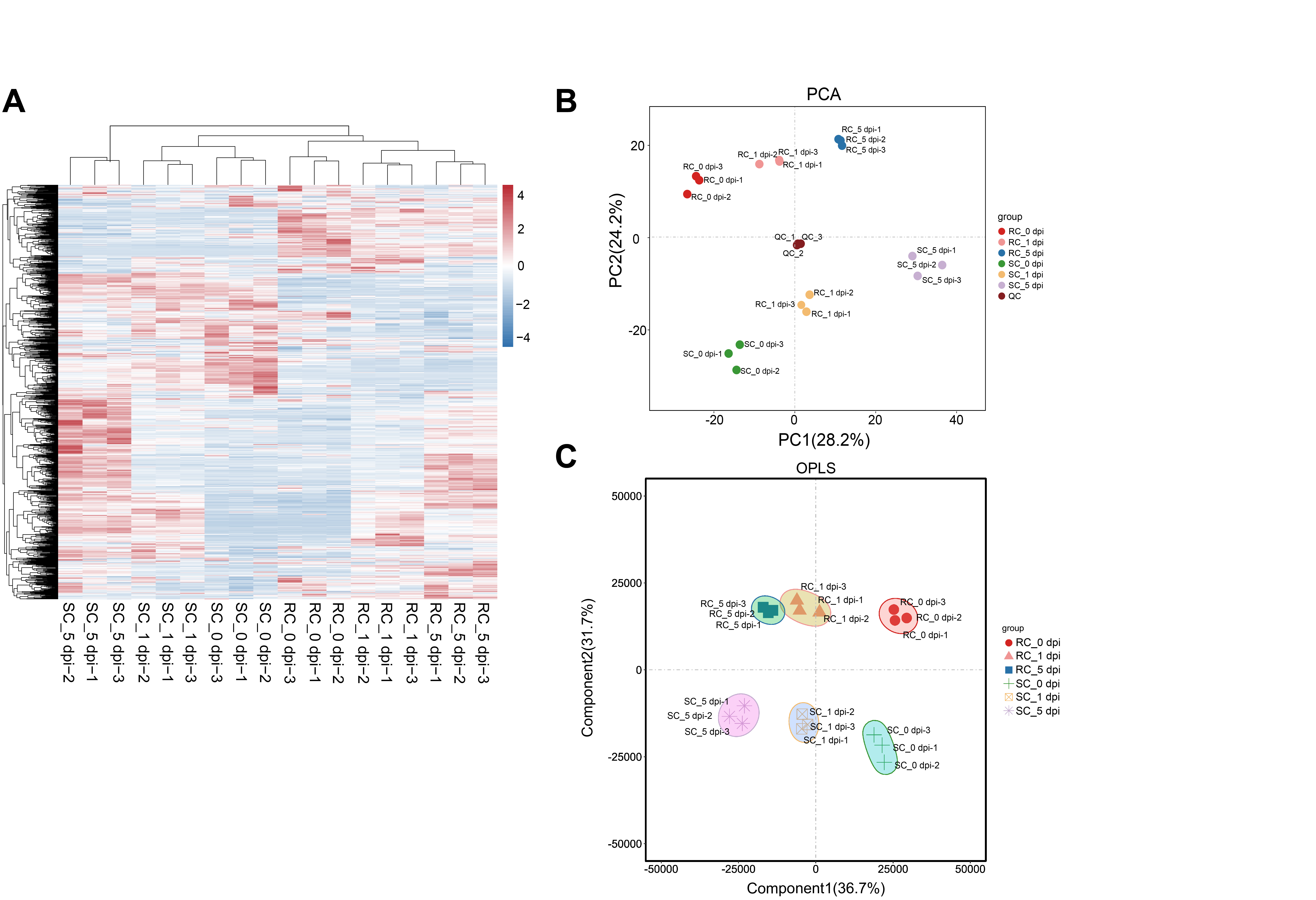


**Figure S3** Quality control of metabolomics data. (A) Heatmap showing the results of the clustering analysis of differentially accumulated metabolites (DAMs). (B) PCA of DAMs. (C) Analysis of DAMs based on OPLS-DA scores.


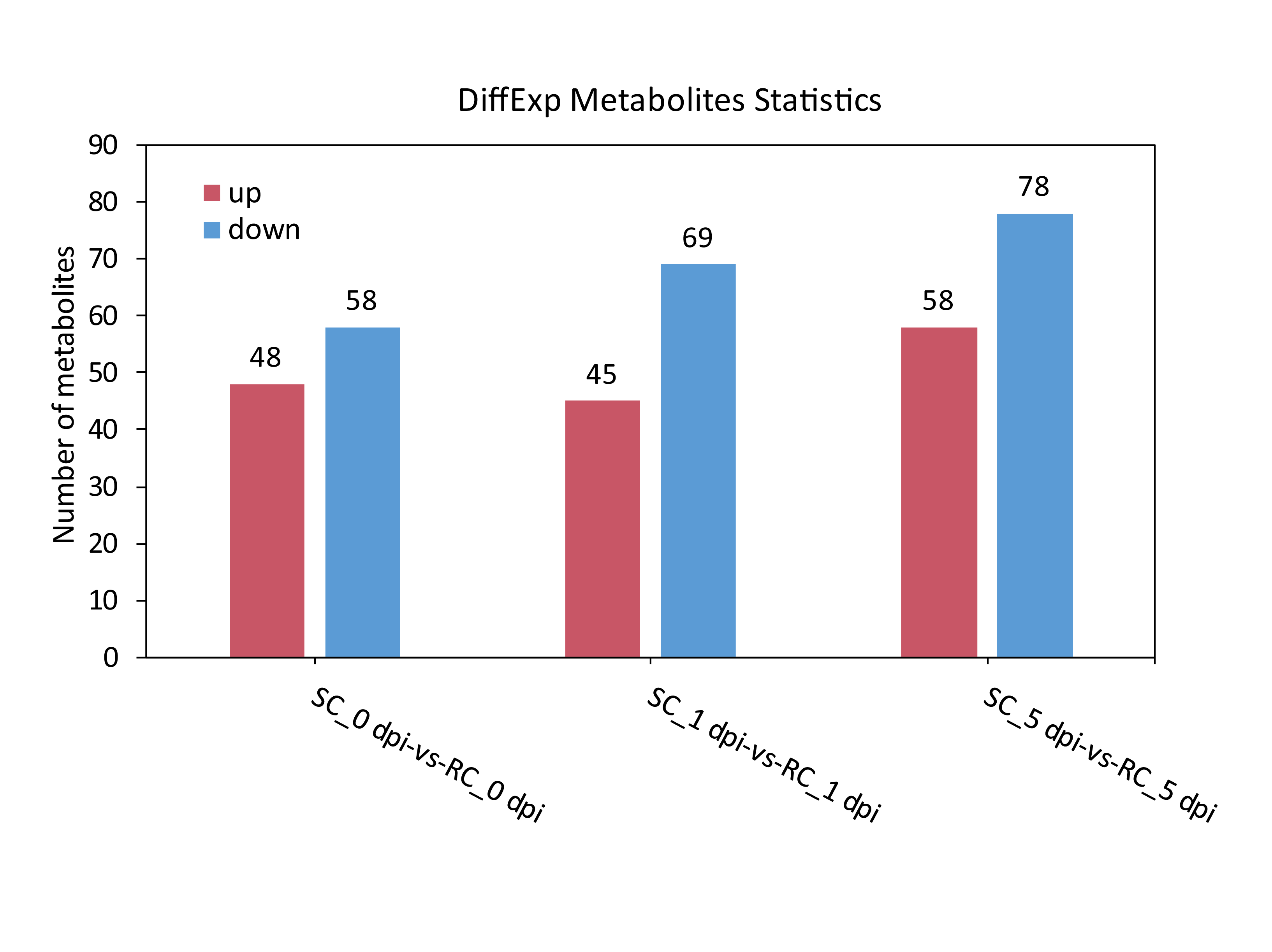


**Figure S4** The number of up- and down-regulated DAMs in SC and RC inoculated with *A. alternata*.


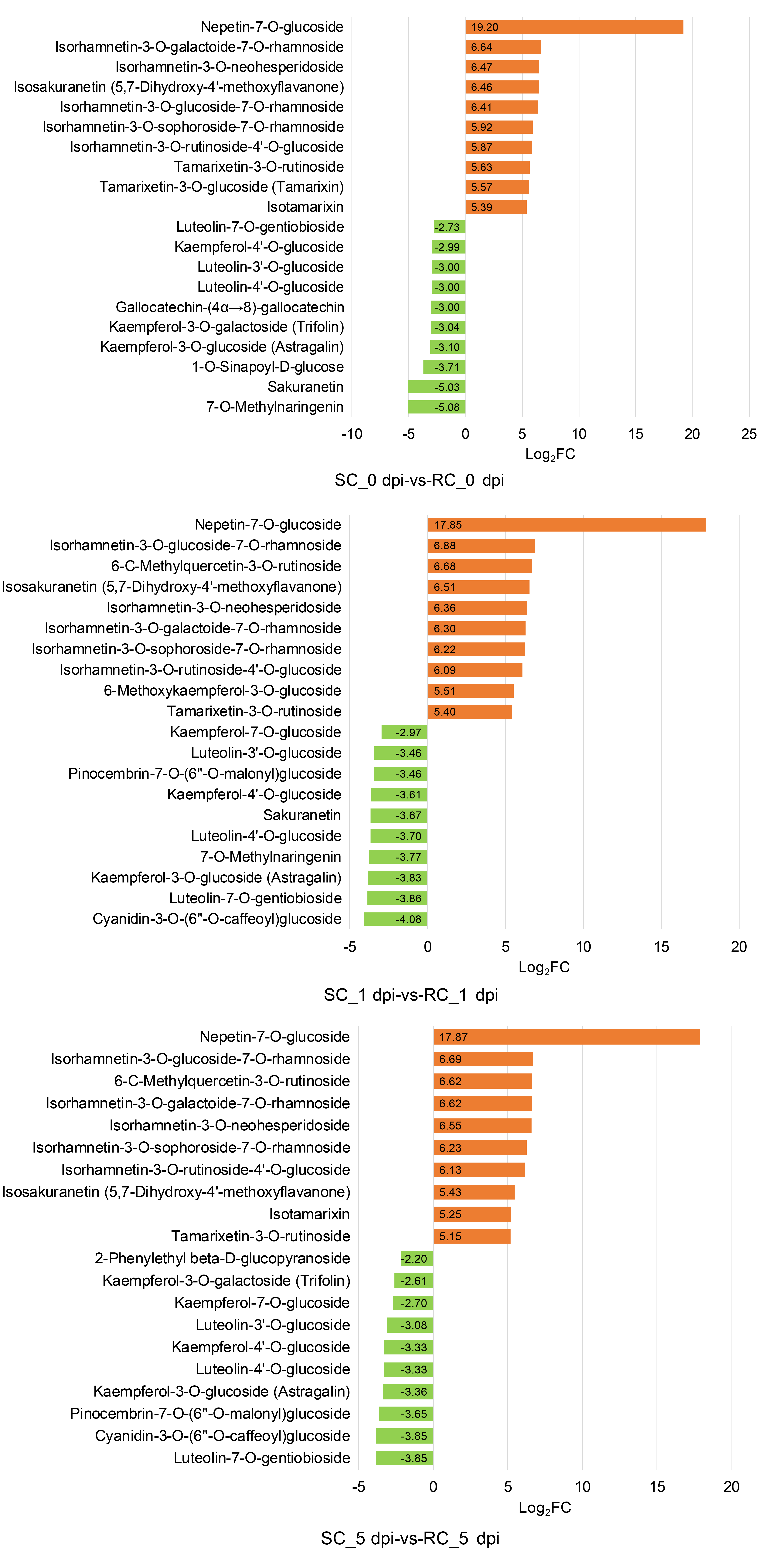


**Figure S5** Up- and down-regulation of the top ten DAMs in SC and RC inoculated with *A. alternata*. The orange and green sections represent up-regulated and down-regulated metabolites, respectively.


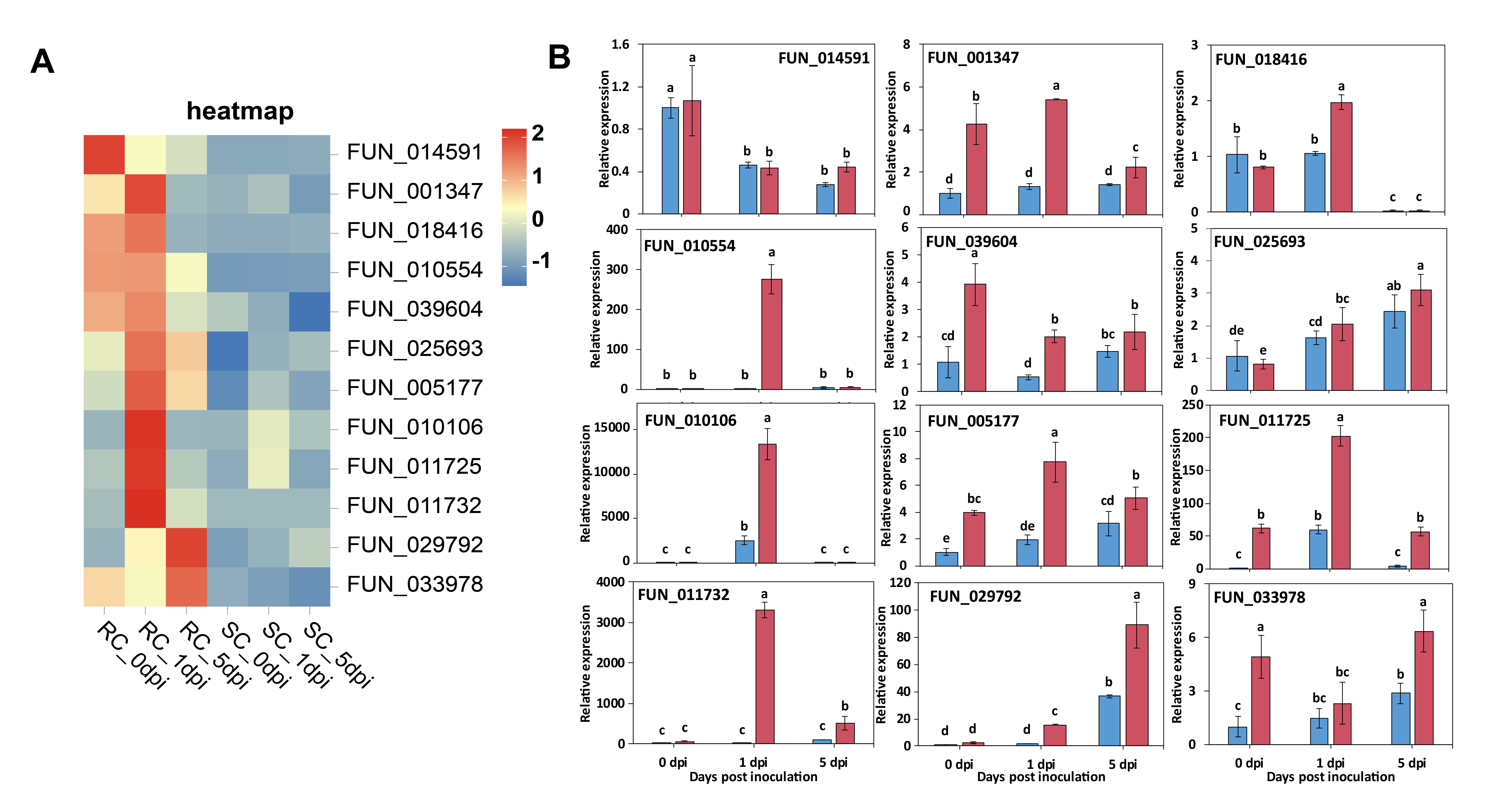


**Figure S6** Confirmation of the expression profiles of select phenylpropanoid biosynthesis pathway genes using RT-qPCR. (A) Heatmaps of DEGs involved in the phenylpropanoid biosynthesis pathway. (B) RT-qPCR analysis to validate the RNA-seq data of phenylpropanoid biosynthesis-related genes. Error bars represent standard deviation (n = 3). Different letters above the bars indicate significant differences at the 0.05 level according to Duncan’s multiple range test.


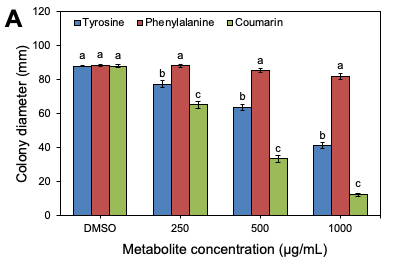

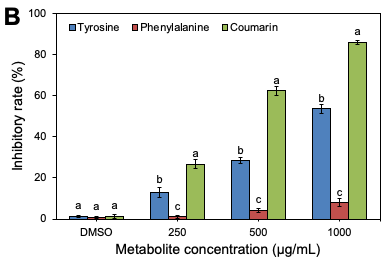


**Figure S7** Effect of the different metabolites on *A. alternata* growth in the plate. (A) Colony diameter of different concentration metabolites against *A. alternata*. (B) Inhibitory rate of different concentration metabolites against *A. alternata*. Error bars represent standard deviation (n = 3). Different letters above the bars indicate significant differences at the 0.05 level according to Duncan’s multiple range test.
